# Robot-assisted laparoscopic management of primary urethra clear cell adenocarcinoma in females: a case report and literature review

**DOI:** 10.3389/fsurg.2026.1865715

**Published:** 2026-09-11

**Authors:** Congwang Chang, Wei Zhang, Hui Shuai, Peng Zhang, Hao Gong, Guanghua Fu, Jianping Liu

**Affiliations:** Department of Urology, The First People's Hospital of Yibin, Yibin, China

**Keywords:** case report, clear cell adenocarcinoma, female, robot-assisted surgery, urethral tumor

## Abstract

**Background:**

Primary clear cell carcinoma of the urethra (CCAU) in women is extremely rare. There are only limited reported cases in the literature, with no standardized treatment protocol presently available. This article aims to explore the clinical characteristics, diagnostic difficulties, treatment strategies, and prognosis of female primary clear cell adenocarcinoma of the urethra, and supplement it with relevant literature analysis to improve the understanding of this rare disease.

**Methods:**

A 57-year-old female patient presented with progressive dysuria for half a year and recurrent urinary retention for 2 months. After evaluation by imaging, endoscopic, and pathological examinations, the patient underwent robot-assisted laparoscopic radical resection of urethral cancer, with the postoperative pathology finally diagnosed as CCAU. Innovative surgical positioning and auxiliary methods were employed during the operation to effectively optimize the treatment and promote the smooth progress of the operation.

**Results:**

Postoperative pathology revealed urethral clear cell adenocarcinoma with sarcomatoid differentiation in some areas. The patient underwent a successful surgical procedure with an uneventful postoperative recovery. Follow-up imaging showed no signs of tumor recurrence. The patient experienced a postoperative complication of urinary incontinence.

**Conclusion:**

CCAU is a rare and highly malignant tumor with poor prognosis. The diagnosis of this disease primarily relies on pathological immunohistochemistry and differential exclusion of metastatic tumors. Multidisciplinary collaboration and radical surgery are key to successful treatment, with consideration given to adjuvant therapy and close postoperative follow-up. At present, no standard surgical regimen exists, and the efficacy of postoperative adjuvant therapy remains unclear.

## Introduction

Primary clear cell carcinoma of the urethra (CCAU) is extremely rare. In 1975, Tiltman first reported a case of clear cell adenocarcinoma in a female patient (1). This disease predominantly affects elderly females, accounting for approximately 0.003% of all female malignant tumors and approximately 0.02% of tumors in the female genitourinary system (2). Because of its rarity and the lack of specific clinical manifestations, diagnosis is often delayed, and no standard treatment currently exists. This article reports a case in which the patient was treated with robot-assisted laparoscopic surgery, and discusses its clinical characteristics, key diagnosis points, and treatment based on the relevant literature.

##  Case report

**Figure 1:**
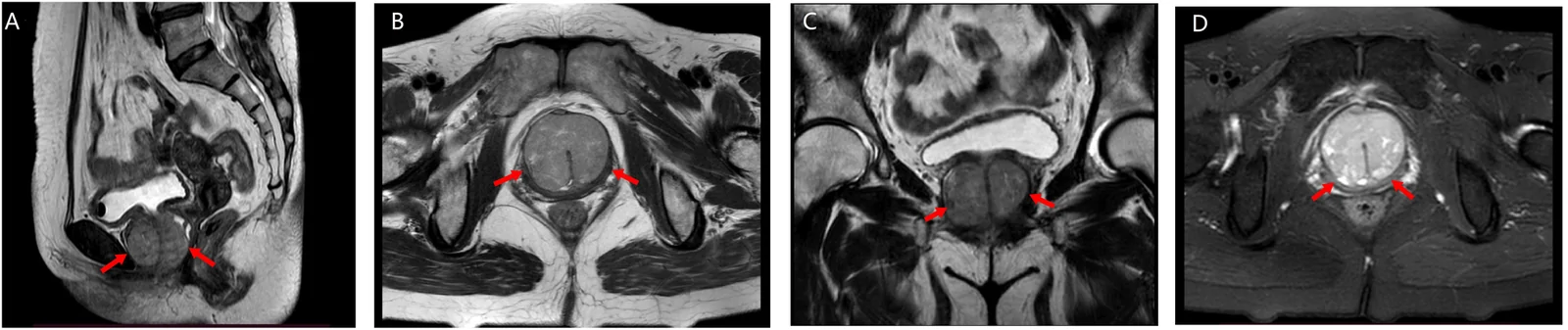
MRI showing a mass, with abnormal signal shadow around the urethra, approximately 4.8 cm × 4.4 cm × 3.9 cm in size, with the tendency to grow around the urethra **(A–C)**. The DWI sequence shows high signal, and the enhanced scan shows mild uneven enhancement, with a shape similar to that of the prostate **(D)**.

**Figure 2:**
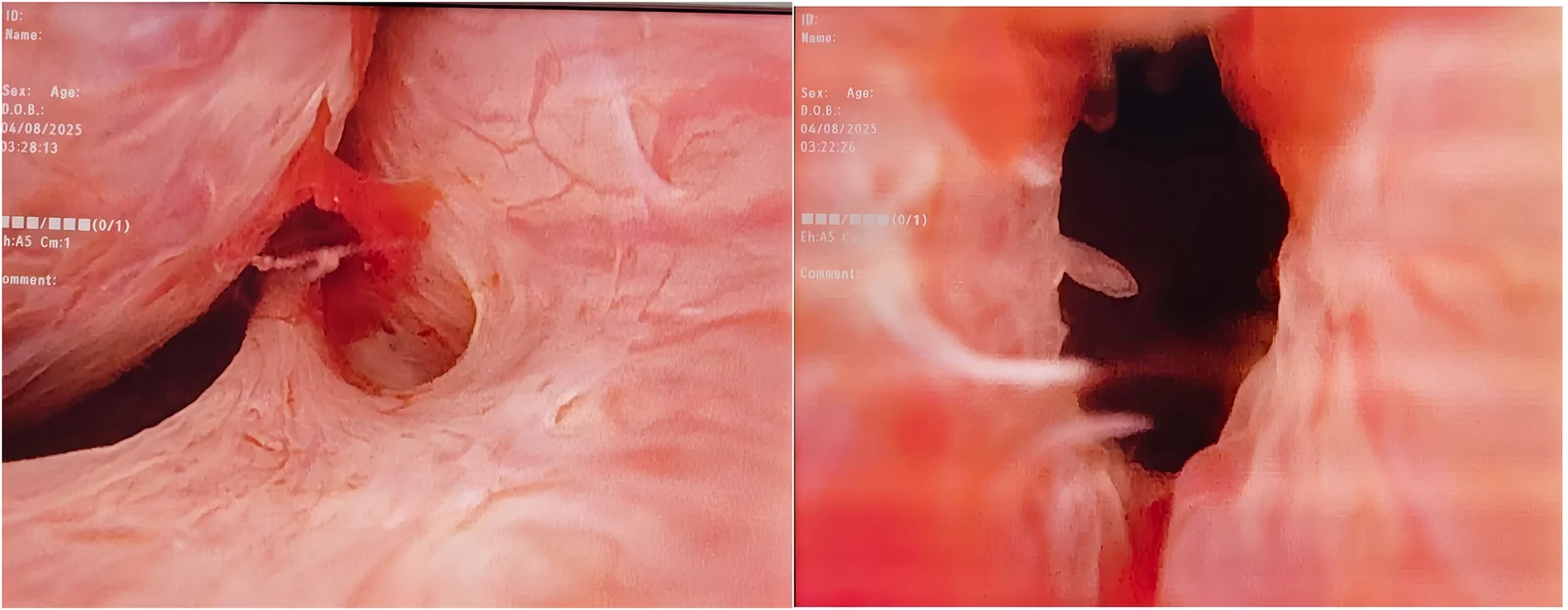
Preoperative urethrocystoscopy.

A 57-year-old female patient was admitted to the hospital due to “progressive dysuria for half a year, accompanied by repeated urinary retention for 2 months,” without symptoms such as frequent urination, urgency of urination, and gross hematuria. Physical examination revealed the following: The external genitalia had developed normally, the vagina was patent, and there was no vaginal discharge. A firm mass measuring approximately 5 cm × 4 cm in diameter could be palpated on the anterior vaginal wall. Routine urinalysis, complete blood count, and liver and kidney function tests showed no abnormalities, while carbohydrate antigen 125 (CA-125) levels were elevated to 173.62 U/mL. Contrast-enhanced magnetic resonance imaging (MRI) of the pelvis showed a mass of abnormal signal shadow around the urethra, approximately 4.8 cm × 4.4 cm × 3.9 cm in size, with a tendency to grow around the urethra. The diffusion-weighted imaging (DWI) sequence showed high signal, and the enhanced scan revealed mild uneven enhancement, with a shape similar to that of the prostate (Figure 1). Pelvic lymph nodes were not enlarged. Preoperative cystoscopic examination showed a urethral lesion suspicious for a prostatic-like neoplasm (Figure 2).

The puncture biopsy of the urethral mass indicated that it was a malignant epithelial tumor. The immunohistochemistry results revealed the following: PAX-8 (+), CK7 (+), P53 (+), PCK (+), Syn (−), CgA (−), CK20 (−), Her-2 (−), GATA-3 (weak +), and the Ki-67 positive rate was approximately 30%. Metastatic cancer and gynecological tumors were preliminarily ruled out. It was considered to be a primary malignant epithelial tumor of the urethra, with clear cell adenocarcinoma being most likely. After multidisciplinary consultation (MDT), the patient underwent robot-assisted laparoscopic radical resection of urethral cancer. Postoperative pathological examination revealed negative margins.

**Figure 3:**
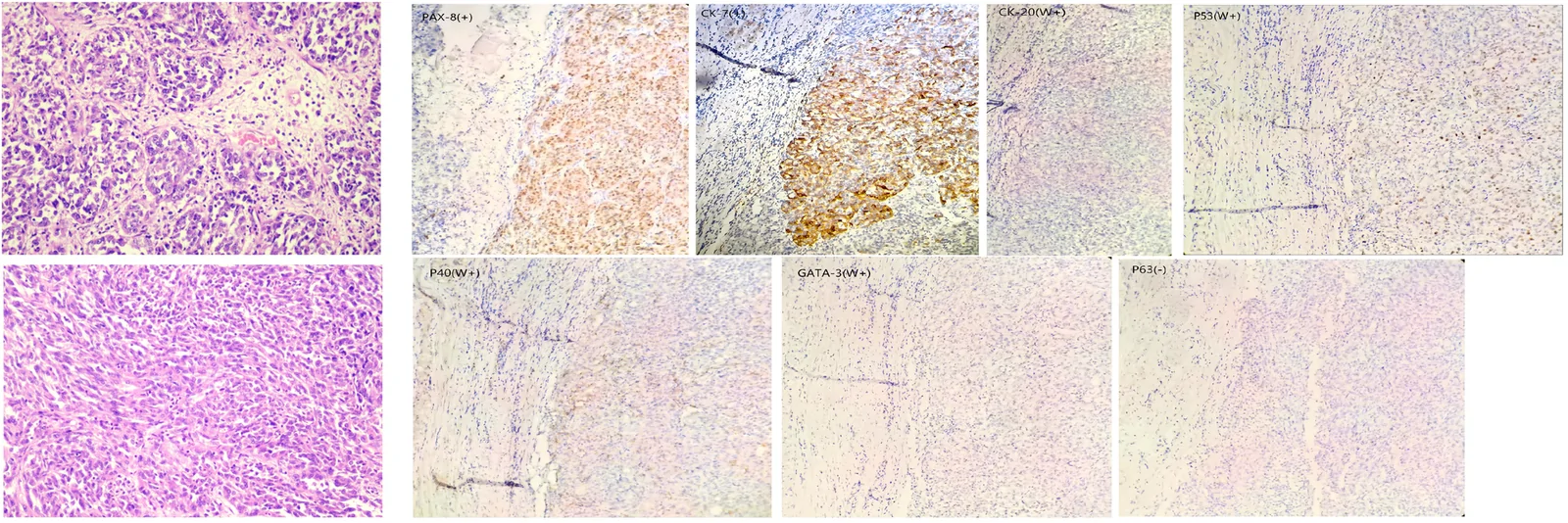
Hematoxylin and eosin staining, with histology and immunohistochemistry findings.

The surgical procedure was based on robot-assisted laparoscopic radical prostatectomy, with innovative modifications including the adoption of the supine lithotomy position with the head lower than the feet, and assistance from an assistant in elevating the anterior vaginal wall during the operation. Finally, the tumor tissue was completely removed; the vagina, uterus, bladder, and external urethral orifice were completely preserved; and the anastomosis between the bladder neck and the external urethral orifice was completed. The final diagnosis of postoperative pathology was “clear cell adenocarcinoma/Müllerian tumor of the urethra, some with sarcomatoid differentiation.” Immunohistochemical results revealed that CK5/6(−), P63(−), and P40(−) suggested that squamous cell carcinoma or partial urothelial carcinoma could be excluded. CK7 (partial +, focal weak +) and CK20 (-) suggested possible urothelium or adenocarcinoma origin. Her-2(−) and GATA-3 (small focal weak +) indicated potential urothelium or breast origin. P53(+, wild type) and PCK(+) suggested epithelial origin. Syn(−), CgA(−), and CD56 (focal weak +) suggested possible non-neuroendocrine tumor. PAX-8(+) strongly suggested a Müllerian origin (such as ovarian or endometrial cancer). However, this manifestation is relatively rare in urethral tumors. Ki67LI(+, about 30%) indicates moderate proliferative activity, which supports the diagnosis of malignancy. PAX-8 positivity raised suspicion for metastatic carcinoma of the female genital tract. However, both the final imaging studies and postoperative pathological findings supported a primary urethral origin of the tumor. Postoperative pathological examination, which included additional testing for NapsinA (focal +) and HNF-1β (weak +), confirmed the diagnosis of clear cell adenocarcinoma (Figure 3).

**Figure 4:**
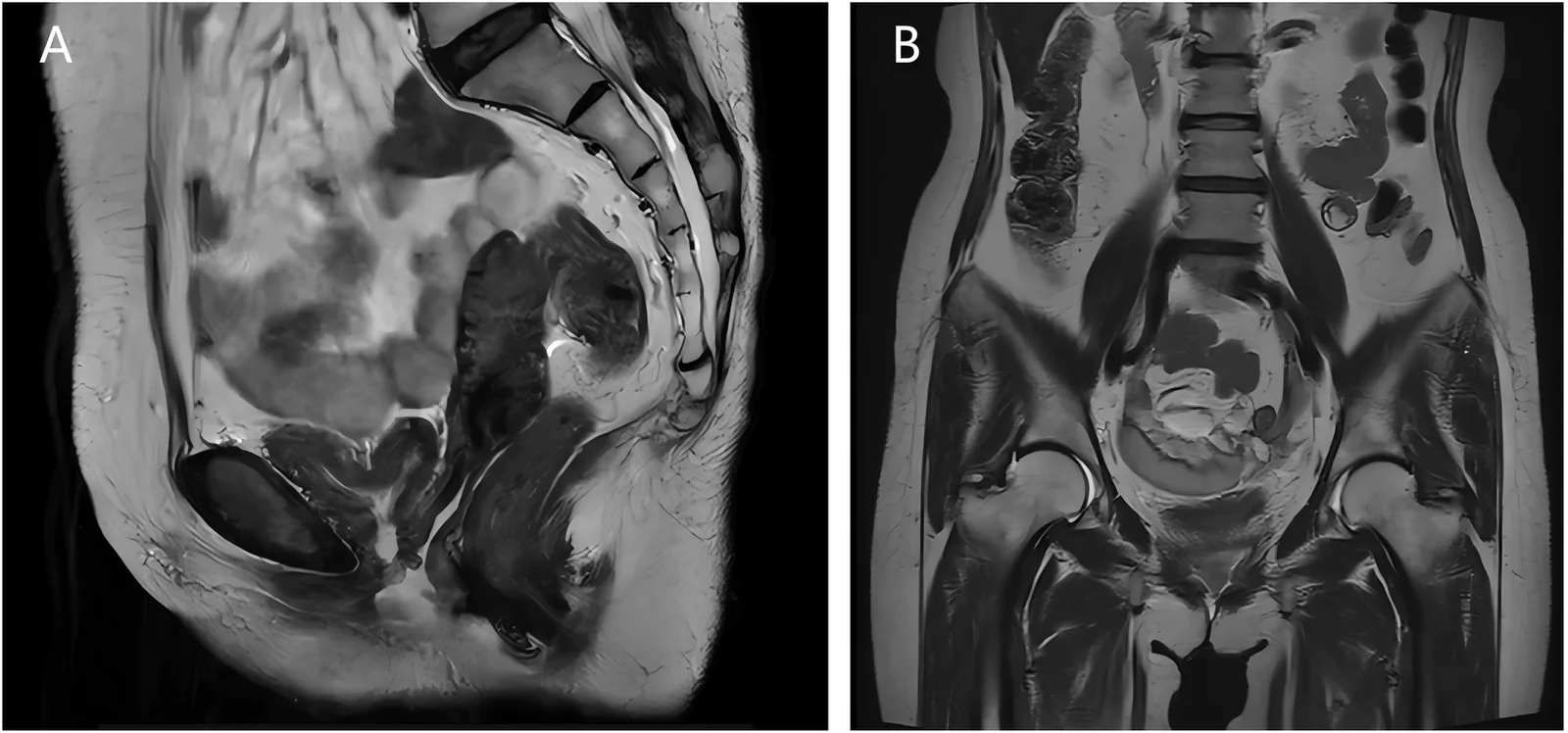
MRI showing no recurrence of the tumor: Sagittal Plane (A), Coronal Plane (B).

The patient's postoperative recovery proceeded smoothly. The urinary catheter was removed two weeks postoperatively, following which the patient developed genuine urinary incontinence, necessitating the long-term use of adult diapers. At the two-month follow-up examination, the carbohydrate antigen 125 levels had returned to normal, and no recurrence of the tumor was detected through pelvic MRI (Figure 4). The patient did not receive any subsequent treatments such as chemotherapy or radiotherapy.

## Discussion

There are currently multiple theoretical hypotheses regarding the origin of CCAU, including originating from Skene's gland or urothelial metaplasia. However, a unified consensus has not yet been reached. Reis et al. proposed and discussed that there exists a type of gland around the female urethra, originating from the urogenital sinus, whose function is similar to that of the male prostate and can secrete prostatic fluid, clinically referred to as the “female prostate” (3).

Dagur et al. further proposed that Skene's gland may be involved in the formation of the female urogenital tract and can be affected by various benign conditions such as inflammation, cysts, and abscesses, as well as affected by malignant lesions including cancer. However, there are still differing opinions regarding this (4). In this case, we opted for preoperative needle biopsy rather than transurethral biopsy, based on preoperative imaging findings showing a predominantly periurethral growth pattern of the tumor. However, needle biopsy may carry a higher risk of tumor seeding along the needle tract. The final diagnosis of CCAU depended on histological and immunohistochemical analysis. It exhibited characteristic microscopic features: The tumor cells were enlarged, the cytoplasm was rich and transparent with obvious vacuoles, the cells were arranged like spikes, and transparent spheroids could be seen. The differential diagnosis of primary urethral clear cell adenocarcinoma includes (1) metastatic clear cell carcinoma from the female genital tract (ovary, endometrium); (2) metastatic renal cell carcinoma; (3) urothelial carcinoma with clear cell features; and (4) nephrogenic adenoma with clear cell change (5). It should be noted that both primary urethral clear cell adenocarcinoma and Leydig–Sertoli collision tumors of the testis represent exceptionally rare genitourinary malignancies that require meticulous clinicopathological correlation for accurate diagnosis. In both entities, the absence of standardized treatment protocols necessitates multidisciplinary evaluation and individualized treatment planning, and both highlight the importance of detailed histopathological and immunohistochemical characterization in guiding clinical management (6).

CCAU is a highly aggressive malignant tumor with an extremely low 5-year survival rate. Even when detected at an early stage, the prognosis remains poor (7). Factors such as age, race, tumor location, tumor size, tumor stage, pathological type, local invasion of the primary tumor, presence of metastasis, and treatment methods are all related to prognosis (8). The clinical symptoms of patients are similar to other types of urethral tumors. Common symptoms include hematuria, urinary frequency, urgency, dysuria, and urinary tract infection (9). Therefore, it cannot be distinguished from other types of urethral tumors through clinical manifestations.

In this case, MRI examination revealed a tumor location and morphology highly similar to those of the male prostate, with no evidence of lymph node metastasis or distant metastasis. Preoperative catecholamine tests were within normal limits, and the total prostate-specific antigen (tPSA) level was less than 0.008 ng/mL. CA-125, a well-established tumor marker, has not been systematically evaluated for its role in primary urethral clear cell adenocarcinoma (5, 10). Possible explanations for the elevation in our patient include tumor production of CA-125 (supported by immunohistochemical positivity), cross-reactivity, or unrelated benign conditions. The postoperative normalization of CA-125 in our case raises the possibility that serum CA-125 could serve as a useful tumor marker for monitoring disease recurrence in CCAU, analogous to its role in ovarian cancer. However, we acknowledge that this remains speculative, and the utility of CA-125 as a surveillance marker in CCAU requires further validation.

Patel et al. conducted a study on prognostic factors in patients with CCAU. The results showed that surgical treatment can improve the overall survival (OS) and disease-free survival (DSS) of patients (11). However, there is still a lack of standard surgical protocols for CCAU. Given the high malignancy and invasive nature of CCAU, surgical resection serves as the primary therapeutic strategy. This approach not only facilitates the excision of existing tumors and alleviation of obstructive symptoms but also aids in pathological sampling (12). The current treatment of primary urethral cancer mainly follows the guidelines first proposed by the European Society of Urology in 2013 (13). For localized early urethral adenocarcinoma, guidelines recommend local tumor resection with preservation of the urethra. In contrast, for advanced-stage paraurethral gland carcinoma in females, a comprehensive treatment approach is advocated, which includes surgical intervention (total pelvic exenteration involving the bladder, uterus, and adnexa, followed by urinary diversion), chemotherapy, and radiotherapy. Based on the patient's condition and personal preference, we opted for robot-assisted laparoscopic radical resection of urethral tumor, without concurrent lymph node dissection or further extended resection. The patient was fully counseled regarding the guideline-recommended approach (urethrectomy with cystectomy and urinary diversion) vs. the alternative of bladder-preserving surgery. She expressed a strong preference for avoiding permanent urinary diversion and its associated impact on quality of life. This preference was respected after multidisciplinary discussion.

Robot-assisted laparoscopy provides magnified three-dimensional visualization and exceptional operational flexibility, demonstrating particularly significant advantages in deep pelvic anatomy dissection and urethral reconstruction. During the operation, robotic assistance provided satisfactory exposure of the operative field. The clear, magnified vision and flexible mechanical arms effectively reduced the technical difficulty of suturing and urethral reconstruction. The patient was positioned supine with the head lower than the feet (Trendelenburg position) during the surgery, effectively exposing the surgical field. However, during the separation of the tumor from the anterior vaginal wall, due to the absence of male anatomical structures such as the seminal vesicles and vas deferens, the exposure of the tumor base was relatively challenging. In previous clinical experiences, it has been common practice to resect the tumor base along with the anterior vaginal wall. However, the surgeon found that the gap between the tumor and the anterior vaginal wall could be clearly exposed by the assistant lifting the anterior vaginal wall with the index finger, so that the tumor could be successfully separated from the anterior vaginal wall, which not only preserved the integrity of the vagina, but also reduced the risk of vaginal fistula after operation. Furthermore, during the anastomosis of the bladder neck and the external urethral orifice, due to the residual urethra being relatively short, it was not feasible to guide the suture through a urinary catheter. However, by having an assistant elevate the external urethral orifice extracorporeally, the external urethral orifice could be clearly exposed, effectively resolving the challenge of difficult suture placement. These technical details may be of practical interest to surgeons managing similar cases.

The extent of surgery in our case was determined by the tumor's limited local extent and the patient’s preference for organ preservation. Due to the scarcity of related cases, it is difficult to compare the long-term functional effects of robot-assisted radical resection of paraurethral glands. Postoperative genuine urinary incontinence is an expected consequence of this procedure, as the urethral sphincteric mechanism is disrupted. In females, the continence mechanism relies on the integrity of the urethral smooth muscle, striated urogenital sphincter, and supporting pelvic floor structures. Resection of a significant portion of the urethra inevitably compromises this mechanism. We discussed with the patient the option of delayed bladder neck reconstruction or other continence procedures (e.g., artificial urinary sphincter, sling procedures) once oncological surveillance confirmed sustained remission.

CCAU is an aggressive malignancy with a relatively poor prognosis, particularly when associated with sarcomatoid differentiation, and recurrence can occur months to years after initial treatment. In this case, the postoperative follow-up period was too short to draw any meaningful conclusions regarding long-term oncological outcomes. Only by analyzing the homogeneous series data of large samples in long-term follow-up can we evaluate this operation more comprehensively.

In retrospect, pelvic lymph node dissection (PLND) could have been considered. However, at the time of surgery, the primary goal was complete local resection with negative margins, and the patient's preference for minimizing surgical morbidity also influenced the decision. We have instituted a rigorous surveillance protocol (imaging every 3–6 months) to detect any nodal or distant recurrence early, at which point systemic therapy or salvage PLND could be considered.

Whether adjuvant radiotherapy and chemotherapy are needed after operation remains controversial. However, based on the high-risk characteristics of tumors (such as large size and sarcomatoid differentiation), most scholars suggest that adjuvant chemotherapy (such as platinum-based chemotherapy) should be adopted after surgery, and local radiotherapy should be considered (14–16). Radiotherapy (external beam radiotherapy or brachytherapy) can be used as an adjuvant treatment modality for early-stage proximal urethral cancer (17).

## Conclusions

The diagnosis of CCAU in females is highly challenging and requires precise confirmation through pathological examination, particularly immunohistochemical techniques. Robot-assisted laparoscopic radical surgery is a safe and effective minimally invasive surgical approach for the treatment of such rare tumors. The long-term prognosis of patients depends on tumor stage, pathological characteristics, and the comprehensiveness of the treatment plan. Therefore, individualized postoperative strategies and long-term follow-up are required.

## Data Availability

The datasets presented in this study can be found in online repositories. The names of the repository/repositories and accession number(s) can be found in the article/Supplementary Material.
